# Subserous Cystic Adenomyosis: A Case Report and Review of the Literature

**DOI:** 10.3389/fsurg.2022.807676

**Published:** 2022-03-31

**Authors:** Tongtong Xu, Yue Li, Lili Jiang, Qifang Liu, Kuiran Liu

**Affiliations:** Department of Obstetrics and Gynecology, Shengjing Hospital of China Medical University, Shenyang, China

**Keywords:** cystic adenomyosis, persistent abdominal pain, massive vaginal bleeding, emergency laparotomy, post-operative pathology

## Abstract

**Introduction:**

Cystic adenomyosis is a rare type of adenomyosis that often occurs in adolescents or women of childbearing age. Due to the few reports of this case, its clinical characteristics have not been clearly established.

**Case Presentation:**

We treated a 32-year-old married patient with cystic adenomyosis that reported persistent abdominal pain and massive vaginal bleeding, so an emergency laparotomy was performed. The intraoperative findings and post-operative pathology proved that the diagnosis was correct. The prognosis of the patient is good, and there is no recurrence within 3 months after surgery.

**Results:**

Surgery is the most effective way to treat cystic adenomyosis. Ultrasound and magnetic resonance are the most effective auxiliary examinations for diagnosing the disease.

**Conclusion:**

Cystic adenomyosis is a sporadic disease. This article summarizes this condition's clinical manifestations, pathological features, diagnosis, treatment, and prognosis by reviewing the existing literature and the case presented in this report. It is noteworthy that early diagnosis and individualized treatment strategies can improve patients' quality of life.

## Introduction

Cystic adenomyosis is a rare type of adenomyosis, and only 46 cases of this disease have been previously reported, including the one presented in this study (Table 1). It is important to consider that there are less cases of subserous cystic adenomyosis that can be asymptomatic or manifest as severe dysmenorrhea, increased menstrual flow, and chronic pelvic pain (13). Due to atypical clinical manifestations and rare conditions, patients who suffer from cystic adenomyosis are often misdiagnosed. The symptoms might be interpreted as signs of other clinical conditions such as subserosal uterine fibroids degeneration. We report the case of a 32-year-old woman who underwent surgical treatment for lower abdominal pain and abnormal uterine bleeding. Intraoperative findings and post-operative pathology suggest a possibility of subserous cystic adenomyosis. The patient had a good prognosis without recurrence during the post-operative follow-up period. In addition, we have also reviewed the existing literature to better explain the aspects of the disease and the most effective treatment when a case is diagnosed.

**Table 1:** Case summary of cystic adenomyosis.

**References**	**Age**	**CP**	**Surgical approach**	**Lesion location**	**AT**	**Outcome**	**Lesion size**	**FUT**
							**(cm)**	
Parulekar (1)	36	Pelvic pain	LTME	AM	NO	?	10	?
Dobashi (2)	43	AUB	Hysterectomy	Uterine cavity	NO	Cure	7	?
Tamura (3)	16	HMF SD	LME	AM	NO	Cure	3	?
Giana (4)	46	?	Hysteroscopic	In the uterine cavity	NO	Cure	?	?
Koga (5)	37	Abnomal pain, HMF	Hysterectomy	AM	?	?	17	?
Takeda (6)	20	SD	LME	AM	GnRH	Cure	3	2 Months
	20	SD	LME	AM	NO	?	2.6	?
Ohta (7)	54	HMF	Radical operation	AM	Chemotherapy	?	10	?
Ho (8)	16	Pelvic pain	Resection of the lesion	AM	?	Cure	?	?
Takeuchi (9)	30	SD pelvic pain	LME	?	No	G2P2	3.5	?
	29	Pelvic pain dyspareunia SD	LME	?	NO	G1P1	3	?
	27	Dyspareunia dysmenorrhea	LME	?	NO	Cure	4.2	?
	20	Pelvic pain dysmenorrhea	LME	?	OCPs	Cure	2.8	?
	30	Dysmenorrhea	LME	?	NO	Cure	3.4	?
	28	Dyspareunia dysmenorrhea	LME	?	OCPs GnRH	Cure	2.5	?
	23	Dysmenorrhea pelvic pain	LME	?	OCPs	Cure	2.8	?
	20	Dysmenorrhea pelvic pain	LME	?	GnRH	Cure	3.4	?
	20	Dysmenorrhea	LME	?	OCPs	Cure	3.4	?
Kriplani (10)	16	SD	LME	AM	NO	Cure	4	?
	18	SD	LME	?	NO	Cure	5	?
	16	SD	LME	AM	NO	Cure	4.5	?
	24	SD	LME	AM	NO	Cure	4	?
Marques (11)	17	SD	NO	AM	OCPs	Relief	1.5	?
Iain (12)	19	SD HMF	LME	AM	GnRH	Cure	?	?
	22	Dysmenorrhea HMF infertility	LME	AM	NO	Cure	?	?
Cucinella (13)	25	SD pelvic pain	LME	AM	GnRH	Cure	4.5	3 Months
Kim (14)	30	SD	LME	AM	NO	Cure	2	?
Calagna (15)	39	Severe abdominal pain	LME	Sub-serosal	?	?	6	?
Koukoura (16)	28	SD	LME	AM	NO	Cure	4	1 Year
Pontrelli (17)	27	Dysmenorrhea HMF pelvic pain	Hysteroscopic	Uterine cavity	NO	Cure	?	?
Mori (18)	67	NO	Radical operation	AM	Chemotherapy	Cancerous	11	16 Months
Manta (19)	20	LAP;SD	Laparotomy-mass excision	AM	NO	Cure	4	?
Dadhwal (20)	23	SD LAP	LME	AM	OCPs	Pregnancy	4	?
	16	LAP;SD	LME	AM	OCPs	Cure	3.5	?
Sun (21)	47	HMF dysmenorrhea	Hysteroscopic	Uterine cavity	NO	Cure	?	?
Fan (22)	36	HMF	Hysteroscopic	Uterine cavity	GnRH	Relieve	4	?
	39	NO	Hysterectomy	Uterine cavity	NO	Cure	5	?
Zhou (23)	45	AUB, SD	LME	ECALATPAI	NO	Relieved	9	?
Zhou (24)	29	SD	HIFU	AM	NO	Cure	2.5	6 Months
	34	SD	HIFU	AM	NO	Cure	3.4	6 Months
	20	SD	HIFU	AM	NO	Cure	4	6 Months
	22	SD	HIFU	AM	NO	Cure	2	6 Months
Zhao (25)	30	SD	LME	AM	GnRH	Cure	5.5	4 Months
Minell (26)	19	Dysmenorrhea pelvic pain	sclerotherapy	AM	OCPs	Relieve	2.6	1 Year
Gomez (27)	65	Constipation and urinary frequency	Radical operation	Adjacent myometrium	NO	Cancerous	7.3	?
This case	32	LAP, AUB	Laparotomy-mass excision	Sub-serosal	GnRH	Cure	10	2 Months

*SD, Severe Dysmenorrhea; LAP, Lower abdominal pain; LME, LPS-mass excision; AM, adjacent myometrium; OCPs, Oral contraceptives; P, Pregnancy; SS, sub-serosal; HMF, heavy menstrual flow; DS, dyspareunia; AUB, Abnormal Uterus Beginner; LME, Laparotomy-mass excision; HIFU, high-intensity focused ultrasound; LTME, Laparotomy-mass excision; CP, Clinical presentation; AT, Adjuvant therapy; FUT, Follow-up time; ECALATPAI, exophytic cystic adenomyosis locate at the posterio uterine isthmus*.

## Case Presentation

A 32-year-old woman referred regular menstrual cycles lasting 7 days with a normal flow and had no previous history of period pains. She was admitted to our hospital for abnormal vaginal bleeding for 2 months and lower abdominal pain for 4 days. The abnormal vaginal bleeding was described as persistent and the amount of blood loss was less than her normal menses. The lower abdominal pain was sudden and progressively worsening which she described as dull pain in nature, located over the lower abdomen, and resolved by changing to side lying position.

Physical examination revealed a well-healed cesarean section scar tenderness on palpation of the lower abdomen. Bimanual examination revealed that a cyst palpable in front of the uterus about 10 cm in diameter. It appeared to be attached to front of the uterus.

Laboratory examination showed red blood cell count of 3.2^*^10∧12/L, hemoglobin of 77 g/L. The C-reactive protein level was 24.4 mg/L.

**Figure 1:**
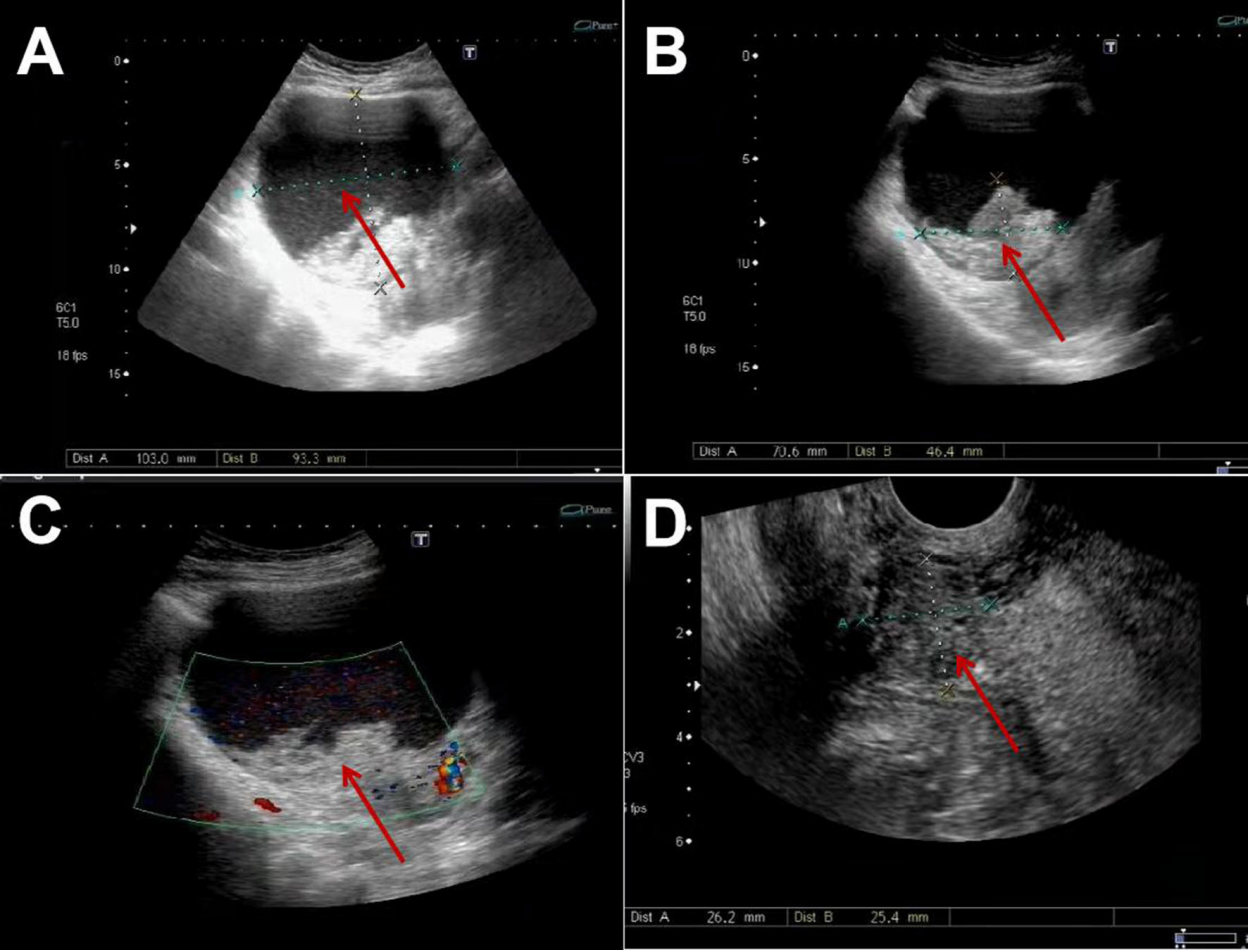
Ultrasonic characteristics of cystic adenomyosis: **(A)** 10.3*10.2*9.3 cm cystic mass can be seen in the front of the uterus. **(B,C)** 7.1*6.5*4.6 cm medium echo mass is seen inside, with clear borders. **(D)** 2.6*2.5*2.5 cm beside it, clear boundary, moderate echo inside, CDFI can detect blood flow signal.

**Figure 2:**
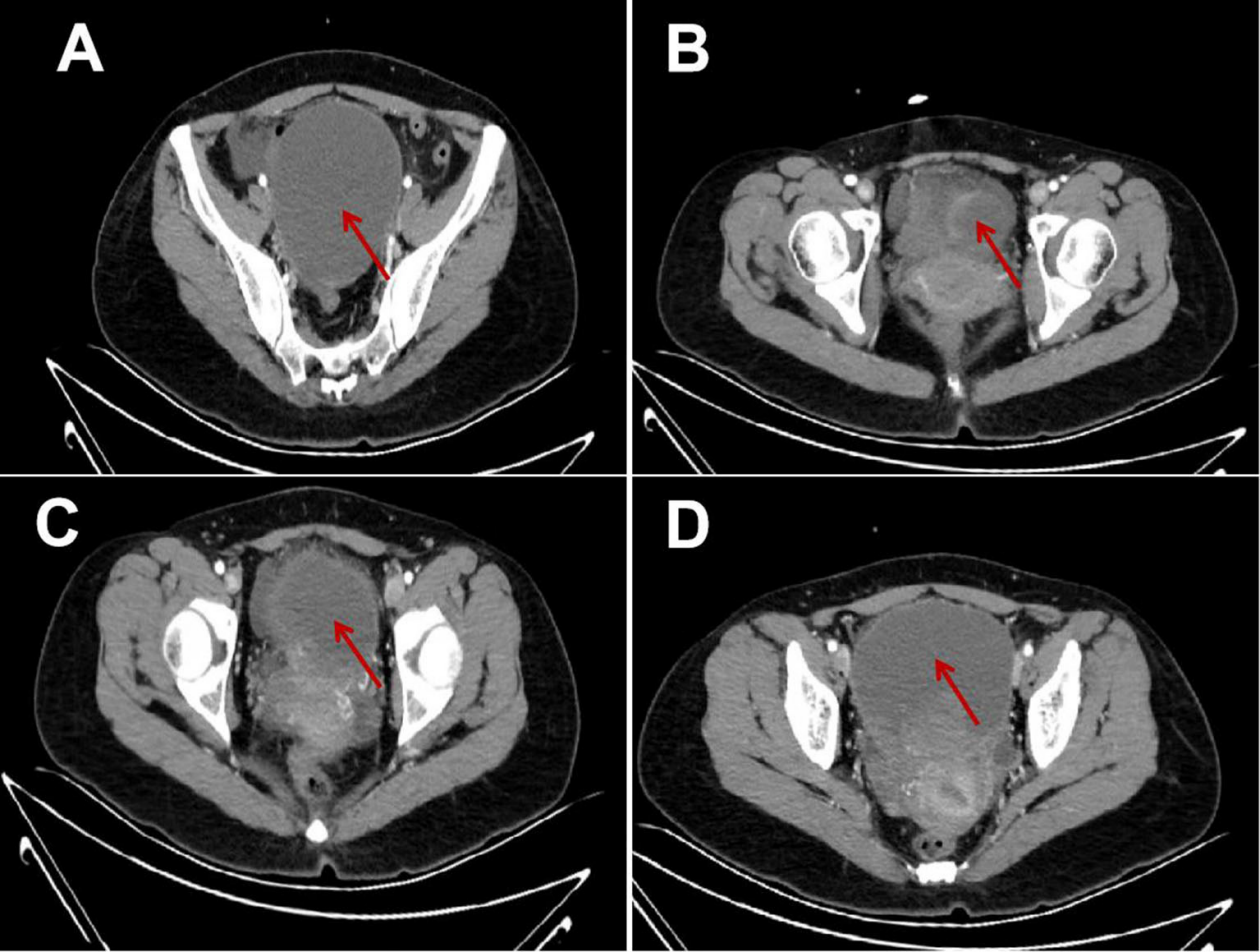
Full-abdominal enhanced CT image of cystic adenomyosis. **(A–D)** the range of cystic masses is about 11.1*9.7 cm, the density is uneven, multiple flocculent high-density shadow deposits can be seen, the enhanced scanning wall is enhanced, and the contents are not enhanced.

Pelvic ultrasound was performed which demonstrated normal adnexae and an enlarged uterus. It revealed a 10.3^*^10.2^*^9.3 cm cystic mass in the front of the uterus (Figure 1A), and a 7.1^*^6.5^*^4.6 cm medium echo mass in the cystic mass with no apparent blood flow signal was detected by CDFI (Figures 1B,C), and 2.6^*^2.5^*^2.5 cm masses could be seen outside the lower part of the anterior wall of the uterus (Figure 1D). The two masses were closely adjacent to the cesarean section incision in the anterior wall of the uterus. The whole abdomen CT scan showed that cystic mass could be seen in the front of the uterus, with a range of about 11.1 ^*^ 9.7 cm. The boundary between the lesion and the anterior wall of the uterus was unclear (Figures 2A–D).

**Figure 3:**
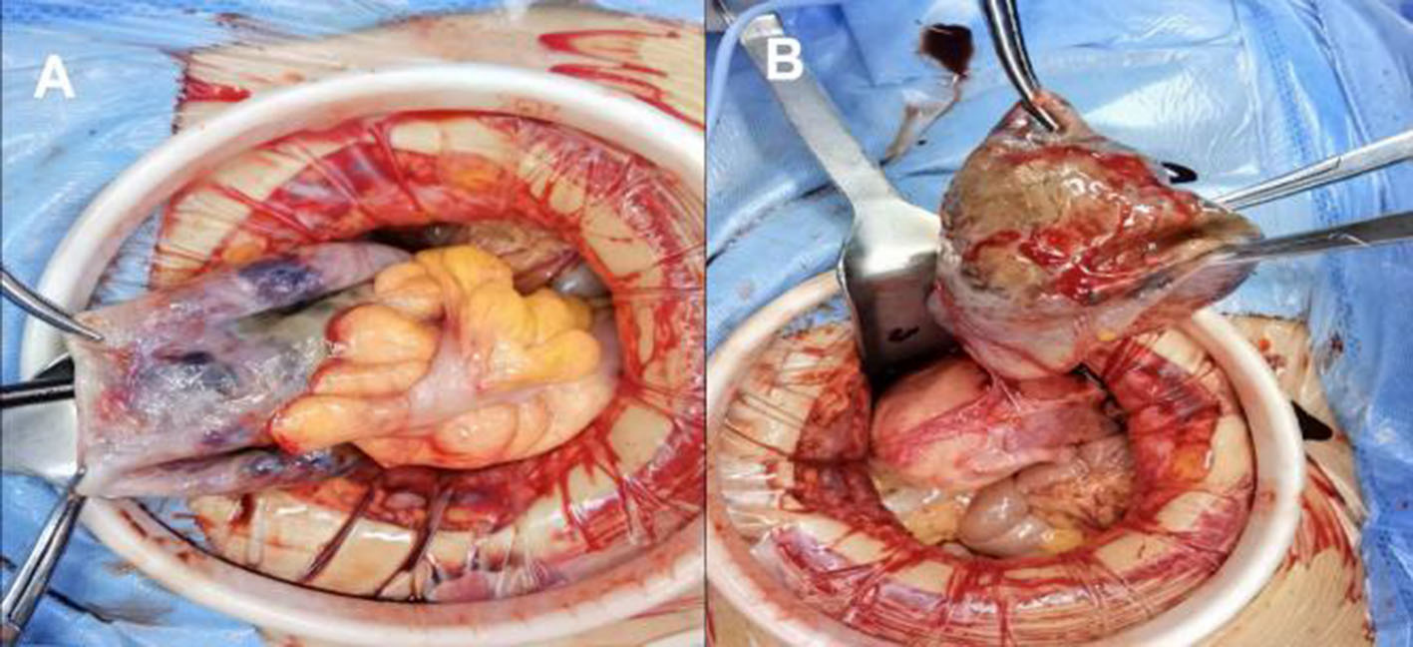
**(A,B)** Intraoperative pictures of patients.

Based on the imaging studies and patient's history, suspicion of cystic adenomyosis was raised. We performed transabdominal surgery on the patient. The intraoperative exploration revealed a cystic mass with a size of about 10^*^10 cm arising from the cervical isthmus of the anterior wall of the uterus. We have detected a chocolate-like viscous liquid inside the cyst and noticed the adhesion of the anterior wall of the cyst to the anterior wall of the uterus and the bladder. The posterior wall of cyst adhered to the intestines, as shown in Figures 3A,B. There was an adenomyoma-like nodule about 3^*^3 cm deep in the cyst. It showed unremarkable adnexae.

**Figure 4:**
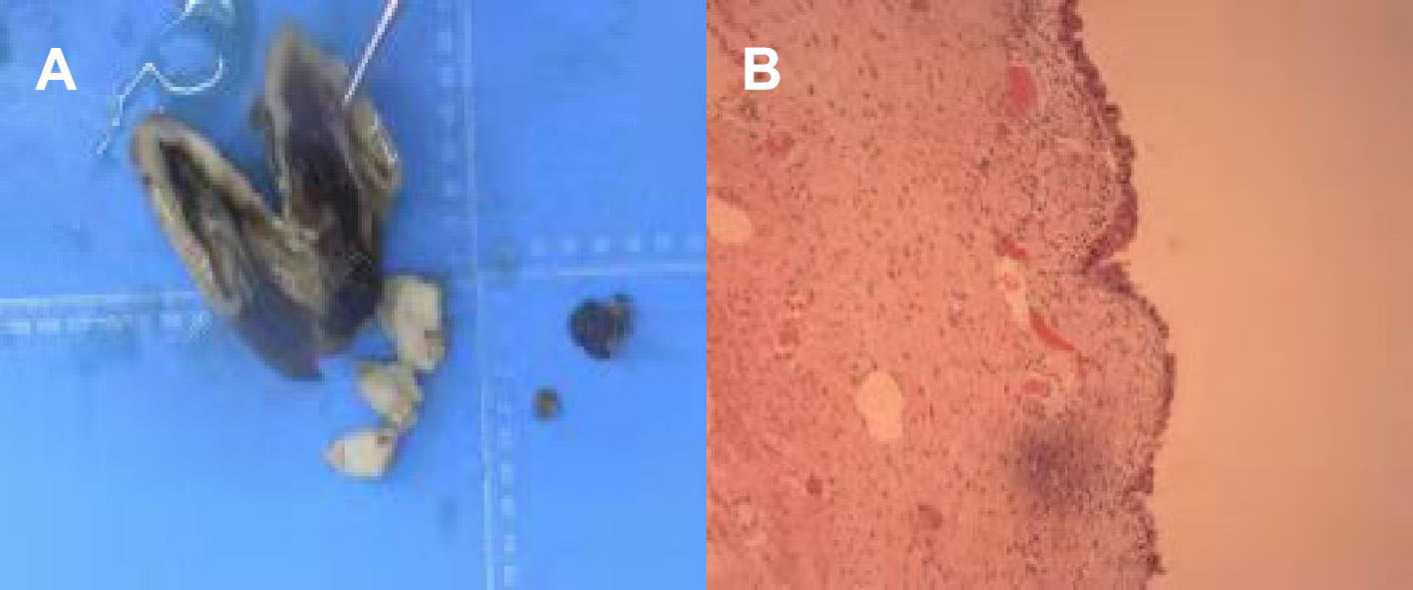
Post-operative pathology pictures, **(A,B)** General view. **(A)** anterior wall cyst of the uterus, about 10 cm in diameter, has been dissected, the contents are lost, part of the inner wall is brown. **(B)** The tissue is covered by the endometrial epithelium, and no abnormality is seen.

She was nursed in the ward for a total of 5 days after the surgery and was discharged well without any post-operative complications. We asked the patient to continue treatment with GnRH after operation. Post-operative pathology confirmed cystic adenomyosis (Figures 4A,B). The final diagnosis was cystic adenomyosis.

**Figure 5:**
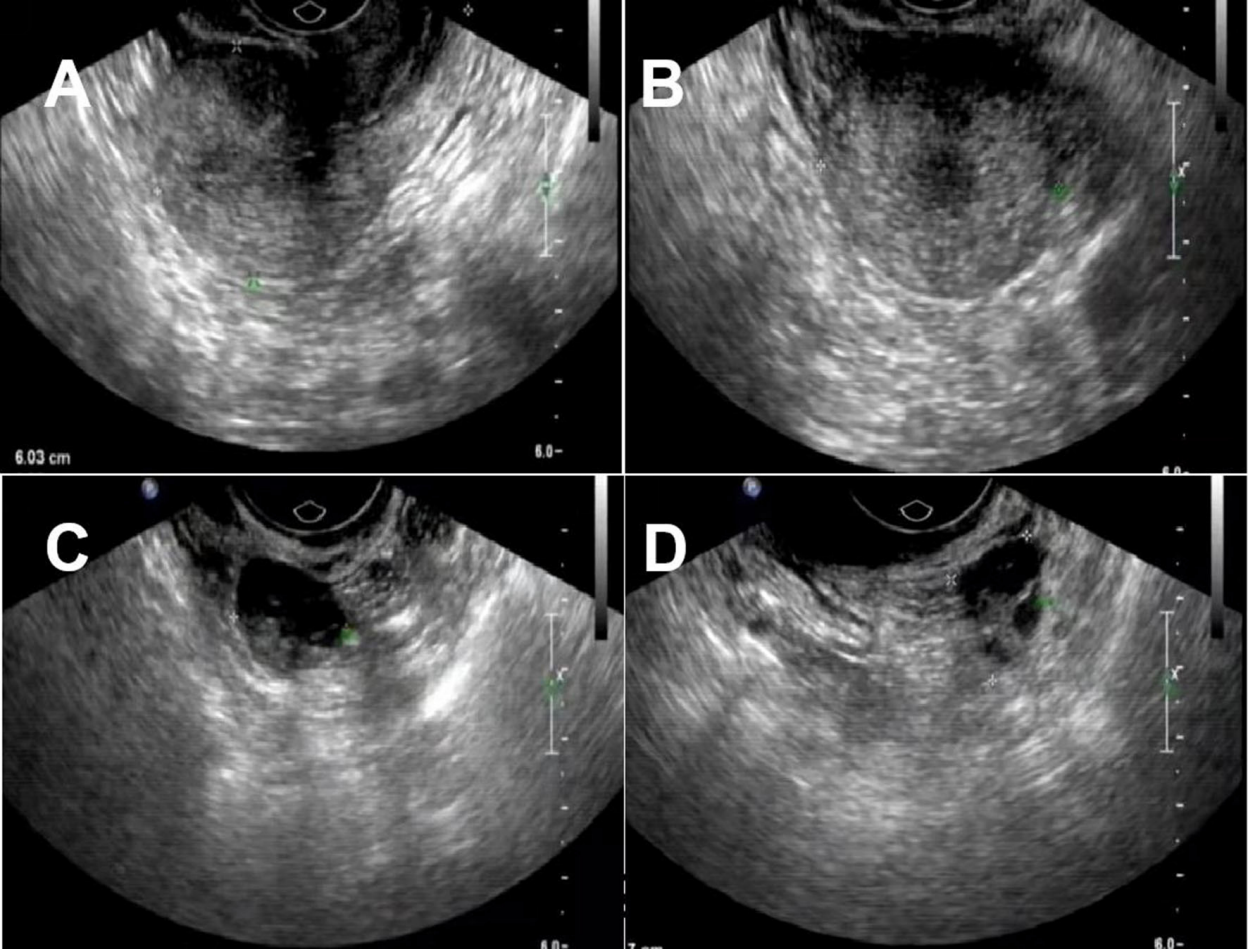
The ultrasound images 2 months after the operation. **(A–D)** No abnormalities in uterus and double appendages.

Two months after operation, we followed up the patient and no abnormality was found in ultrasonography (Figures 5A–D). The patient remains asymptomatic.

## Discussion

Adenomyosis is a common gynecological disease in gynecology caused by the infiltration of the myometrium by the glands and stroma of the endometrium (28). According to the scope of the lesion, it can be divided into diffuse and focal adenomyosis. Diffuse adenomyosis is the most common type, but focal adenomyosis, especially on the cystic uterine glands, is rare (19). Cystic adenomyosis is a particular type of lesion that originates from the myometrium of the uterus filled with a chocolate-like viscous liquid. So far, only 46 cases of cystic adenomyosis have been reported, including our patient (Table 1). According to the location of the cyst in the uterine wall, cystic adenomyosis is divided into five subtypes (29). The A1 subtype includes submucosal or intramural cystic adenomyosis; the A2 subtype includes cystic polypoid lesions; the B1 subtype includes subserosal cystic adenomyosis; the B2 subtype includes cases of intrauterine growth; and the C subtype includes similar cysts in the uterus. Most cystic adenomyosis occurs in the myometrium, and, in a few cases, the lesion is located under the serous membrane or submucosa of the uterine.

The first case of cystic adenomyosis was reported by Parulekar (1), and the medical community has been accumulating knowledge of this disease since then. According to the age of patients, cystic adenomyosis can be divided into two categories: juvenile (primary type) and adult cystic adenomyosis (secondary type) (16). Juvenile cystic adenomyosis generally occurs 5 years after menarche or in patients around 18 years old, while adult cystic adenomyosis generally occurs in patients over 30. In 1996, Tamura et al. (3) reported the first case of juvenile cystic adenomyosis, but its pathogenesis is still unclear. Some professionals interpret it as a congenital disease since some parts of the Mullerian duct are damaged during the development process, causing these parts not to degenerate in time during the development process. After menarche, the remaining Mullerian epithelial cells periodically bleed under the action of estrogen to form cysts. On the other hand, juvenile cystic adenomyosis can also be interpreted as a latrogenic disease when patients have a history of different types of uterine surgery before the onset. In these cases, clinical manifestations often appear after the history of uterine surgery. The clinical symptoms of cystic adenomyosis are non-specific, including dysmenorrhea, chronic pelvic pain, and dysfunctional uterine bleeding that begin early before or after menarche. Dysmenorrhea tends to worsen gradually and is resistant to painkillers or periodic oral contraceptives. Among these 46 cases, most of the patients reported increased menstrual flow and severe dysmenorrhea, and one of them suffered from infertility issues. Three patients presented abnormal uterine bleeding, three haddyspareunia, and two had no clinical symptoms, but the examination revealed the diagnosis. Generally, the diameter of the cysts ranges from 1.5 to 17 cm (refer to Table 1 for details).

Due to its atypical symptoms, cystic adenomyosis should be clearly distinguished from subserosal uterine fibroids degeneration, ovarian endometriosis, and uterine malformations. The diagnosis mainly depends on ultrasound procedures, magnetic resonance, and other auxiliary examinations (30). Cystic adenomyosis lesions showed high signal intensity on the T1-weighted image and significantly low signal intensity on the T2-weighted image (31). Therefore, laboratory tests such as CA-125 can be used as pre-operative diagnostic indicators and one of the dynamic monitoring indicators for post-operative follow-up. Takeuchi et al. (9) reported that the patient's CA-125 level could be normal or >500 U/mL. The diagnostic criteria for cystic adenomyosis are as follows (25): (1) isolated lesions; (2) no abnormalities in the uterus, fallopian tubes, and ovaries; (3) post-operative lesions with pathological reports of cystic adenomyosis; (4) excised lesions with endometrial glands and interstitium; (5) lesions that contain thick chocolate-like liquid; and (6) small lesions of adenomyosis, such as adenomyoma, that can be seen in the muscle layer adjacent to the lesion.

Cystic adenomyosis directly affects patients' quality of life but, more importantly, some may lead to a secondary malignant transformation that can be life-threatening. Therefore, it is essential to take active and effective treatment measures when a patient is diagnosed. The treatment mainly depends on the patient's age, the severity of symptoms, the size of the cyst, and other features. The first steps are to remove the lesion, improve fertility, and prevent a recurrence. Drug treatment is suitable for patients with mild symptoms and small lesions. Some drugs routinely used in the clinical treatment of adenomyosis are all suitable for cystic adenomyosis, including gonadotropin, releasing hormone agonists, or contraceptives. Other treatments, such as non-steroidal anti-inflammatory drugs (NSAIDs) can also be a means to relieve the symptoms of dysmenorrhea. However, drug administration only temporarily relieves the symptoms, and the condition is likely to relapse after ceasing the treatment, which is not ideal. Furthermore, oral drugs might be ineffective for younger patients with evident symptoms that can affect fertility, so surgery is still considered the most effective method to treat the disease (11). Lesions can be removed with open surgical resection, laparoscopic surgery, and hysteroscopic surgery (4, 6, 9, 32).

For patients with no fertility requirements, a total hysterectomy can be performed. For patients with specific fertility requirements, resection is the best option. Whenever possible, doctors may opt for minimally invasive procedures, e.g., laparoscopy, hysteroscopy, which will help patients recover fast after surgery. Hysteroscopy is suitable for submucosal cystic adenomyosis, which protrudes in the uterine cavity but it is not ideal for larger cysts. There are still cases treated with traditional open surgery, mainly because it is difficult to diagnose the disease before, and it can be challenging to determine the location and nature of the lesion. Xiao-Jing et al. (24) proposed that the use of high-intensity focused ultrasound to treat cystic adenomyosis is a safe and feasible method. It effectively target the lesion, less traumatizing, and only causes mild adverse reactions. This method does not require a long hospital stay, and it guarantees the integrity of the structure and function of the uterus, so it does not affect fertility. However, the data from their article was collected based on only four cases, so there's a lack of big data to support the effectiveness of this approach.

Considering the 46 cases retrieved to support this study, most patients underwent laparoscopic surgery to remove the lesions. Three patients with larger lesions underwent hysterectomy, four underwent hysteroscopic surgery, four underwent high-intensity ultrasound-focused treatment, and one underwent sclerotherapy. One very young patient was treated with oral contraceptives because the clinical symptoms were not so evident. However, three patients underwent radical surgery for malignant transformation of cystic adenomyosis. Two of them received post-operative adjuvant chemotherapy, and one refused post-operative adjuvant therapy. Except for three cases of malignant transformation, most of the other patients were completely cured, and three of them gave birth after the operation. We chose to adopt traditional open abdominal exploratory surgery in our patient to guarantee the complete removal of the lesion. We combined this method with a post-operative adjuvant GnRH drug treatment and achieved remarkable results. Follow-up after operation revealed that the patient recovered well, and the cyst did not recur. We show the diagnosis and treatment process of the patient in the form of tables (Table 2).

**Table 2:** Patient diagnosis and treatment process.

**Time**	**Clinical symptoms**	**Therapeutic method**	**Supplementary examination**
July 18, 2021	Vaginal bleeding lower abdominal pain	Treated in other hospital without treatment	A cystic mass in the pelvic cavity in front of the uterus (rupture of uterus?)
July 22, 2021	Vaginal bleeding lower abdominal pain	Open surgery, uterine lesion resection	Cystic mass in front of uterus, about 10 cm in diameter. HGB: 77 g/L
July 23, 2021	Post-operative incision pain	Blood transfusion	HGB:67 g/L
September, 2021	NO	GnRH	No abnormality was found in ultrasonic examination

## Conclusion

Cystic adenomyosis is a less common type of adenomyosis that can also manifest as subserous cystic adenomyosis in some rare cases. Taking into consideration the previous findings about this condition, its clinical manifestations are atypical. Some patients may have no corresponding clinical symptoms, or many manifest the disease with other related symptoms such as dysmenorrhea and abnormal uterine bleeding. Pelvic ultrasound, magnetic resonance, and CA-125 examination can assist in diagnosing this disease, but the final call still depends on intraoperative exploration and post-operative paraffin pathology. Therefore, it can be challenging to effectively diagnose cystic adenomyosis patients before surgery. When another patient has symptoms of abdominal pain and vaginal bleeding, imaging examination shows cystic mass in front of the uterus, we should think that it may be cystic adenomyosis.

This condition can reduce the quality of life of patients, and, in some cases, lead to secondary cancer, which is more life-threatening. Among the 46 cases officially reported previously by other scholars, only three patients with malignant transformation underwent radical surgery and post-operative adjuvant chemotherapy. Therefore, timely diagnosis and treatment is particularly important. In future clinical work, we should comprehensively understand and learn the disease's diagnosis and treatment plan to improve the clinical cure rate. This is the best way to guarantee that patients can receive timely and effective treatment, so we can upgrade their quality of life and post-operative pregnancy rates.

## Data Availability Statement

The original contributions presented in the study are included in the article/supplementary material, further inquiries can be directed to the corresponding author.

## Ethics Statement

Written informed consent was obtained from the individual(s) for the publication of any potentially identifiable images or data included in this article.

## Author Contributions

TX completed most of the writing work. YL and TX reviewed the literature and participated in the drafting of the manuscript. LJ and QL prepared radiographic images. KL was responsible for revising the manuscripts of important knowledge content. All authors gave final approval to the version to be submitted.

## Conflict of Interest

The authors declare that the research was conducted in the absence of any commercial or financial relationships that could be construed as a potential conflict of interest.

## Publisher's Note

All claims expressed in this article are solely those of the authors and do not necessarily represent those of their affiliated organizations, or those of the publisher, the editors and the reviewers. Any product that may be evaluated in this article, or claim that may be made by its manufacturer, is not guaranteed or endorsed by the publisher.
